# Man’s Average Height

**Published:** 1878-02

**Authors:** 


### Selections.

### MAN'S AVERAGE HEIGHT.

Statistical tables taken by army surgeons during the war show the mean height of over a half million men of different nativities. From these it appears the American Indian stands at the head of the list, his mean height being 67.934 inches. The American white man is next in order, being 67.672 inches. The Scotch 67.066. English 66.575. Russia 66.393. France 66.227. Mexico 66.110.—*Exchange.*

